# Supplementary material for: Manipulating midbrain dopamine neurons and reward-related behaviors with light-controllable nicotinic acetylcholine receptors
Source: eLife. 2018 Sep 4;7:e37487. doi: 10.7554/eLife.37487 (PMC6122951; doi:10.7554/eLife.37487)
Supplement: Transparent reporting form [file elife-37487-transrepform.docx]

***eLife’s* transparent reporting form**

We encourage authors to provide detailed information *within their submission* to facilitate the interpretation and replication of experiments. Authors can upload supporting documentation to indicate the use of appropriate reporting guidelines for health-related research (see [EQUATOR Network](http://www.equator-network.org/%20)), life science research (see the [BioSharing Information Resource](https://biosharing.org/)), or the [ARRIVE guidelines](http://www.plosbiology.org/article/info:doi/10.1371/journal.pbio.1000412) for reporting work involving animal research. Where applicable, authors should refer to any relevant reporting standards documents in this form.

If you have any questions, please consult our Journal Policies and/or contact us: [editorial@elifesciences.org](mailto:editorial@elifesciences.org).

**Sample-size estimation**

- You should state whether an appropriate sample size was computed when the study was being designed
- You should state the statistical method of sample size computation and any required assumptions
- If no explicit power analysis was used, you should describe how you decided what sample (replicate) size (number) to use

Please outline where this information can be found within the submission (e.g., sections or figure legends), or explain why this information doesn’t apply to your submission:

**In methods section “Statistical analysis” (p 27):**

“No statistical methods were used to predetermine sample sizes, which are comparable to many studies using similar techniques and animal models.”

**In all figure legends:**

For each experiment, sample sizes (e.g. number of cells, number of mice) and p-values are indicated in the corresponding figure legend and, when possible, on the graph (e.g. barplots).

**Replicates**

- You should report how often each experiment was performed
- You should include a definition of biological versus technical replication
- The data obtained should be provided and sufficient information should be provided to indicate the number of independent biological and/or technical replicates
- If you encountered any outliers, you should describe how these were handled
- Criteria for exclusion/inclusion of data should be clearly stated
- High-throughput sequence data should be uploaded before submission, with a private link for reviewers provided (these are available from both GEO and ArrayExpress)

Please outline where this information can be found within the submission (e.g., sections or figure legends), or explain why this information doesn’t apply to your submission:

**Methods, Section “Animals” p17**

65 Wild-type male C57BL/6J mice were obtained from Janvier Laboratories (France) and 6 knockout SOPF-HO-ACNB2 (β2-/-) male mice were obtained from Charles Rivers Laboratories (France).

**Methods, Section “Chronic guide cannula implantation” p24**

“Following stereotaxic viral infection in the VTA (as described above), 14 WT C57BL/6J mice were implanted with a chronic opto-fluid guide cannula (Doric Lenses Inc, Canada, see Fig 4A) using the same coordinates. “

**In all figure legends:**

For each experiment, sample sizes (e.g. number of cells, number of mice) and p-values are indicated in the corresponding figure legend and, when possible, on the graph (e.g. barplots).

Detailed sample sizes are given below:

**Fig 1E**

9 eGFP+ cells

**Fig 1F**

5 eGFP+ cells

**Fig 2C-E**

6 neurons from 2 WT mice, treated with the photoswitch

10 eGFP+ neurons from 8 WT mice injected with the virus, and treated with the photoswitch

**Fig 3B-G**

Controls 28 neurons from 4 WT mice injected with the photoswitch

93 neurons from 10 WT mice injected with the virus and the photoswitch

**Fig 4B-D**

7 neurons from 6 WT mice injected with the virus and the photoswitch

**Fig 5C-E**

Saline group: 6 WT mice

Nicotine groups: 15 (7+8) WT mice

β2^-/-^ group: 6 SOPF-HO-ACNB2 mice

Opto-pharmacology groups: 14 (7+7) WT mice injected with the virus and the photoswitch

Note: 15(7+8) indicates that experiment has been done twice and data were pooled.

**Statistical reporting**

- Statistical analysis methods should be described and justified
- Raw data should be presented in figures whenever informative to do so (typically when N per group is less than 10)
- For each experiment, you should identify the statistical tests used, exact values of N, definitions of center, methods of multiple test correction, and dispersion and precision measures (e.g., mean, median, SD, SEM, confidence intervals; and, for the major substantive results, a measure of effect size (e.g., Pearson's r, Cohen's d)
- Report exact p-values wherever possible alongside the summary statistics and 95% confidence intervals. These should be reported for all key questions and not only when the p-value is less than 0.05.

Please outline where this information can be found within the submission (e.g., sections or figure legends), or explain why this information doesn’t apply to your submission:

**In methods section “Statistical analysis” (p 27):**

“No statistical methods were used to predetermine sample sizes. Data are plotted as mean ± SEM. Total number (n) of observations in each group and statistics used are indicated in figure and/or figure legend. Unless otherwise stated, comparisons between means were performed using parametric tests (two-sample t-test) when parameters followed a normal distribution (Shapiro test P > 0.05), and non-parametric tests (here, Wilcoxon or Mann-Whitney (U-test)) when this was not the case. Homogeneity of variances was tested preliminarily and the t-tests were Welch-corrected if needed. Multiple comparisons were Holm-Bonferroni corrected. Comparison between the cumulative distributions of *in vivo* multi-unit recordings between controls and LinAChRs (Fig. 3D) was performed using a Kolmogorov-Smirnov test. P > 0.05 was considered to be not statistically significant.”

**In all figure legends:**

For each experiment, sample sizes (e.g. number of cells, number of mice) and p-values are indicated in the corresponding figure legend and, when possible, on the graph (e.g. barplots).

(For large datasets, or papers with a very large number of statistical tests, you may upload a single table file with tests, Ns, etc., with reference to sections in the manuscript.)

**Group allocation**

- Indicate how samples were allocated into experimental groups (in the case of clinical studies, please specify allocation to treatment method); if randomization was used, please also state if restricted randomization was applied
- Indicate if masking was used during group allocation, data collection and/or data analysis

Please outline where this information can be found within the submission (e.g., sections or figure legends), or explain why this information doesn’t apply to your submission:

**In methods, Section “Nicotine-induced place preference paradigm” p24 :**

“Pretest data were used to segregate the animals with equal bias so each group has an initial preference score almost null, indicating no preference on average. On day 2, 3 and 4, animals received an i.p. injection of nicotine tartrate (0.5 mg/kg, in PBS) or an equivalent injection of saline (PBS), and immediately confined to one of the pairing chambers for 1200 s (20 min). The CPP test was performed using a single nicotine concentration (0.5 mg/kg) which is known to induce preference in mice. Groups were balanced so the animals do not always get nicotine in the same chamber. On the evening of the same day, mice received an injection of the alternate solution (nicotine or saline) and were placed in the opposite pairing chamber. The saline control animals received a saline injection in both pairing compartments.”

**Additional data files (“source data”)**

- We encourage you to upload relevant additional data files, such as numerical data that are represented as a graph in a figure, or as a summary table
- Where provided, these should be in the most useful format, and they can be uploaded as “Source data” files linked to a main figure or table
- Include model definition files including the full list of parameters used
- Include code used for data analysis (e.g., R, MatLab)
- Avoid stating that data files are “available upon request”

Please indicate the figures or tables for which source data files have been provided:

For a better understanding, we included a supplementary material containing 5 additional figures and corresponding legends. All data generated or analyzed during this study are included in the manuscript and supporting files. Source data have been provided for Figures 1-5.
